# Evaluating the effects of safety incentives on worker safety behavior control through image-based activity classification

**DOI:** 10.3389/fpubh.2024.1430697

**Published:** 2024-08-12

**Authors:** Bogyeong Lee, Hyunsoo Kim

**Affiliations:** ^1^Department of ICT Integrated Ocean Smart Cities Engineering, Dong-A University, Busan, Republic of Korea; ^2^Department of Architectural Engineering, Dankook University, Yongin, Republic of Korea

**Keywords:** construction safety incentive program, safety regulations, activity recognition, computer-vision, spatiotemporal graph convolutional network

## Abstract

**Introduction:**

Construction worker safety remains a major concern even as task automation increases. Although safety incentives have been introduced to encourage safety compliance, it is still difficult to accurately measure the effectiveness of these measures. A simple count of accident rates and lower numbers do not necessarily mean that workers are properly complying with safety regulations. To address this problem, this study proposes an image-based approach to monitor moment-by-moment worker safety behavior and evaluate the effects of different safety incentive scenarios.

**Methods:**

By capturing workers’ safety behaviors using a model integrated with OpenPose and spatiotemporal graph convolutional network, this study evaluated the effects of safety-incentive scenarios on workers’ compliance with rules while on the job. The safety incentive scenarios in this study were designed as 1) varying the type (i.e., providing rewards and penalties) of incentives and 2) varying the frequency of feedback about ones’ own compliance status during tasks. The effects of the scenarios were compared to the average compliance rates of three safety regulations (i.e., personal protective equipment self-monitoring hazard avoidance, and arranging the safety hook) for each scenario.

**Results:**

The results show that 1) rewarding a good-compliance is more effective when there is no feedback on compliance status, and 2) penalizing non-compliance is more effective when there are three feedbacks during the tasks.

**Discussion:**

This study provides a more accurate assessment of safety incentives and their effectiveness by focusing on safe behaviors to promote safety compliance among construction workers.

## Introduction

Worker safety in the construction industry should not be overstated, even though many tasks have recently been automated, and the number of workers on the site is gradually decreasing. Automated processes partially simplify the work process, and these efforts can lead to a reduction in accidents due to human error (1, 2). However, the number of fatal accidents among construction workers is still high (3–6). Although there are several causes of accidents at construction sites, most occur because workers violate minor safety rules (7–9). Previous studies have suggested several interventions that can motivate workers to comply with safety regulations in their tasks (10–14). Workers are offered incentives (i.e., financial or business benefits) for safe behavior if they comply with their safety regulations. Existing studies have validated the effects of policies by counting the frequency of accidents (15–17). Thus, the accident rate is considered as the result of violating safety regulations, and the decreasing accident rate can prove the positive effect of safety incentives. However, this approach cannot ensure that accidents are entirely prevented due to safety incentives. This implies the possibility of an overly optimistic assessment of incentive policies in cases where workers do not comply with safety standards despite the absence of accidents. For a more accurate assessment of safety incentives, it is necessary to evaluate whether such policies induce a behavior change (i.e., compliance with safety regulations) among workers, regardless of the accident rate.

However, measuring behavior change in the context of safety is challenging. To assess whether incentive measures are effective, one must observe whether the relevant measures lead to changes in worker behavior, such as workers being aware of and complying with safety regulations in their tasks. It is difficult to measure these behavioral changes, especially at subtle levels such as increased awareness (e.g., checking or avoiding the hazards). Many studies have confirmed the effectiveness of safety incentive policies, however, have explored them over a relatively long term, such as a few months. Though evaluating incentive programs on a long-term basis is a valid approach for capturing safety behavior, not only the effect of incentive but also the environmental factors of a worksite, safety climate, and group effect of workers can affect the evaluation results. It is required to investigate only focusing on an individual’s safety behavior and the effect of the safety incentive program on behavioral change.

To address these problems, the objective of this study is to capture the moment-by-moment safety behavior of workers under different safety incentive scenarios and evaluate the effects of the corresponding incentive policies. We used an image-based approach to observe workers’ momentary behaviors and their changes. Recently, several researchers (18–26) have developed an image-based approach to detect workers’ behavior using image data collected from construction sites within a certain timeframe. Considering that the human body can be recognized as a skeleton in an image, with sequential data, this study captures the safe behavior of workers using OpenPose and recognizes whether they perform their safe behavior correctly. We designed safety incentive scenarios that differ in the type of incentives [i.e., type (rewards/penalties) and the timing of compliance status]. During experiments with subjects experiencing different incentive scenarios, we evaluated the effects of each scenario by examining subjects’ compliance with safety regulations for each task. The results were compared to the subjects’ average compliance rates for the five incentive scenarios. This study makes a significant contribution of the literature because construction worker safety remains a major concern even as task automation increases. Though safety incentives have been introduced to encourage safety compliance, it is still difficult to accurately measure the effectiveness of these measures. The approach used in this study to determine worker compliance with safety regulations can easily capture momentary safe behaviors and improve existing incentive programs that typically focus on outcomes by focusing more on the safe behaviors themselves.

## Related works

### Effects of reward and penalty policies on worker safety behavior

Incentive policies in the workplace can improve workers’ task-related behaviors, especially by increasing labor productivity (27–29) and promoting safe behaviors (30–33). Safety incentive programs (SIP) have been introduced, especially in the construction and manufacturing industries, where worker safety is critical (12, 16). To motivate workers to comply with safety regulations without relaxing them, various types of incentive programs have been developed, including financial benefits and personnel appraisals (31, 34–36). Existing studies have confirmed that there is a positive effect on worker safety behavior and investigated which way of providing incentives has higher effects (37–40). Most of them (32, 37, 41, 42) find that financial benefits have larger effects than other types of compensation, even if the magnitude of the effect steadily decreases over time. In terms of how incentives are provided, related studies (34, 38, 43) have compared the influences of incentive and disincentive (i.e., penalty) schemes. The differences between the two were caused by the responses of the corresponding workers’ to positive rewards and negative punishments. According to prospect theory (44–46), a person tends to behave in a more risk-averse manner when their compliance behavior guarantees a reward. A counterargument (36, 47), is that an expected punishment encourages compliance with the rules due to fear of loss. Even when individual differences are taken into account, deterrents have a greater effect on worker compliance with safety regulations in most cases. For example, imposing a penalty may create the impression that workers do not want to incur a loss, and it raises alarms among workers.

Similarly, incentives and penalties may also be an important mechanism influencing incentives for safety behavior. Previous research (34, 36, 48, 49) has found that the severity, certainty, and speed of punishment are related to the effects of penalties on rule compliance in the context of information technology security and driving behavior. Empirical studies (34, 39, 40, 50) have also found that severity and certainty do not proportionally affect rates of rule compliance. Thus, incentive and penalty policies can be effective when attention and empirical learning are present where appropriate rewards and penalties occur.

There are two types of incentive policies for safety behavior: (1) Outcome-Based Safety Incentive Program (OBSIP), and (2) Behavior-Based Safety Incentive Program (BBSIP) (32, 51, 52). These two differ in whether the incentives are based on the outcome or the behavior itself. In the case of construction work, the behavior itself should be more critical than the outcome in evaluating worker compliance. For example, the absence of accidents does not necessarily guarantee compliance with safe behaviors on a construction site. It is rare that non-compliance with safe behavior turns out to be an accident directly, as in many cases, it results in a near miss with some luck (53–55). Therefore, it is critical to determine workers’ adherence to safe behaviors rather than outcomes, because the primary objective of encouraging workers to adhere to safe behavior is to fundamentally prevent accidents through their compliance.

To confirm the effectiveness of reward and punishment mechanisms in compliance with construction work safety rules in an actual working environment, it is necessary to define strategies that vary the intensity and frequency of rewards and punishments, and to measure the compliance rate according to each strategy. To this end, this study requires a model that can identify compliance with momentary safety behaviors that occur in a relatively short period of time (i.e., 1–3 s) and provide feedback to the corresponding workers on compliance results.

### Determining worker compliance with safety regulations using sequential images

The safety behavior required during construction work typically consist of sets of continuous moment-by-moment actions. As an example of self-checking for personal protective equipment (PPE), behaviors include raising and lowering the workers’ arms of to tap their safety helmet in a specific sequence, even if the detailed action will vary for each individual. These safety regulations should be performed repeatedly in the short term between tasks or according to the random locations of hazards during workers’ working hours. To determine worker compliance with safe behavior and provide feedback, a monitoring method that can continuously track worker behavior is needed. The image-based human activity recognition approach has been widely used in construction (18, 19, 22, 56) and the performance of the approach has been radically improved for practical use. Recently, human activity or object recognition based on image data obtained from video recordings at a construction site has been used to verify worker safety (20, 24, 26, 56–58) and work progress (59–62).

Among the numerous deep-learning-based algorithms for recognizing objects from visual data, recent studies have used the spatiotemporal graph convolutional network (ST-GCN), which can recognize a series of actions as an activity (63, 64). By detecting the pose per frame based on the corresponding joints and skeletons through OpenPose (65, 66) and using this information as input, the ST-GCN detects the activity while considering the temporal sequence of multiple actions. This approach is widely used in the medical field (67–69) to compare a patient’s gait (e.g., Parkinson’s disease) with that of a healthy person, or to evaluate a patient’s gait over time to track the progression of a disease. Other cases include assessing posture during exercises, such as yoga, to self-detect incorrect postures using video-recordings (70, 71). Because this approach can consider the position of joints and skeleton in successive frames and the temporal sequence of poses simultaneously, it can measure the compliance or compliance level of the activity compared to the correct criterion of the moment-by-moment activities. In addition, it allows automatic verification of behavioral compliance regardless of the duration or frequency of the behaviors. Taking advantage of this, a previous study (20) implemented the identification of typical safety regulations in construction activities to determine whether workers properly comply with the corresponding regulations. The study found that this approach can maximize the efficiency of safety monitoring, which needs to capture multiple behaviors performed sporadically by multiple workers at different locations on a construction site. Based on reliable performance in the construction work environment (20), this approach can be used as a basis for providing feedback to workers or implementing safety incentives by measuring rate of safety compliance by individual workers.

## Methodology

To motivate workers to comply with safety regulations at their workplaces, various safety incentives can be created. To evaluate the effectiveness of the incentives, we measured the change in compliance rate after the introduction of the corresponding incentive scenarios. Because the change in compliance rate can directly reflect a change in behavior, this study designed various safety incentive scenarios and compared the compliance rate of each scenario to evaluate the effects of the safety incentive measures. To measure the compliance rate of each subject, a model that could identify the compliance status of each safe behavior from the recorded video was used by incorporating the OpenPose and ST-GCN algorithms. Because numerous workers are simultaneously working on a construction site, monitoring their safe behaviors requires an automatic method to detect many behavior changes.

**Figure 1:**
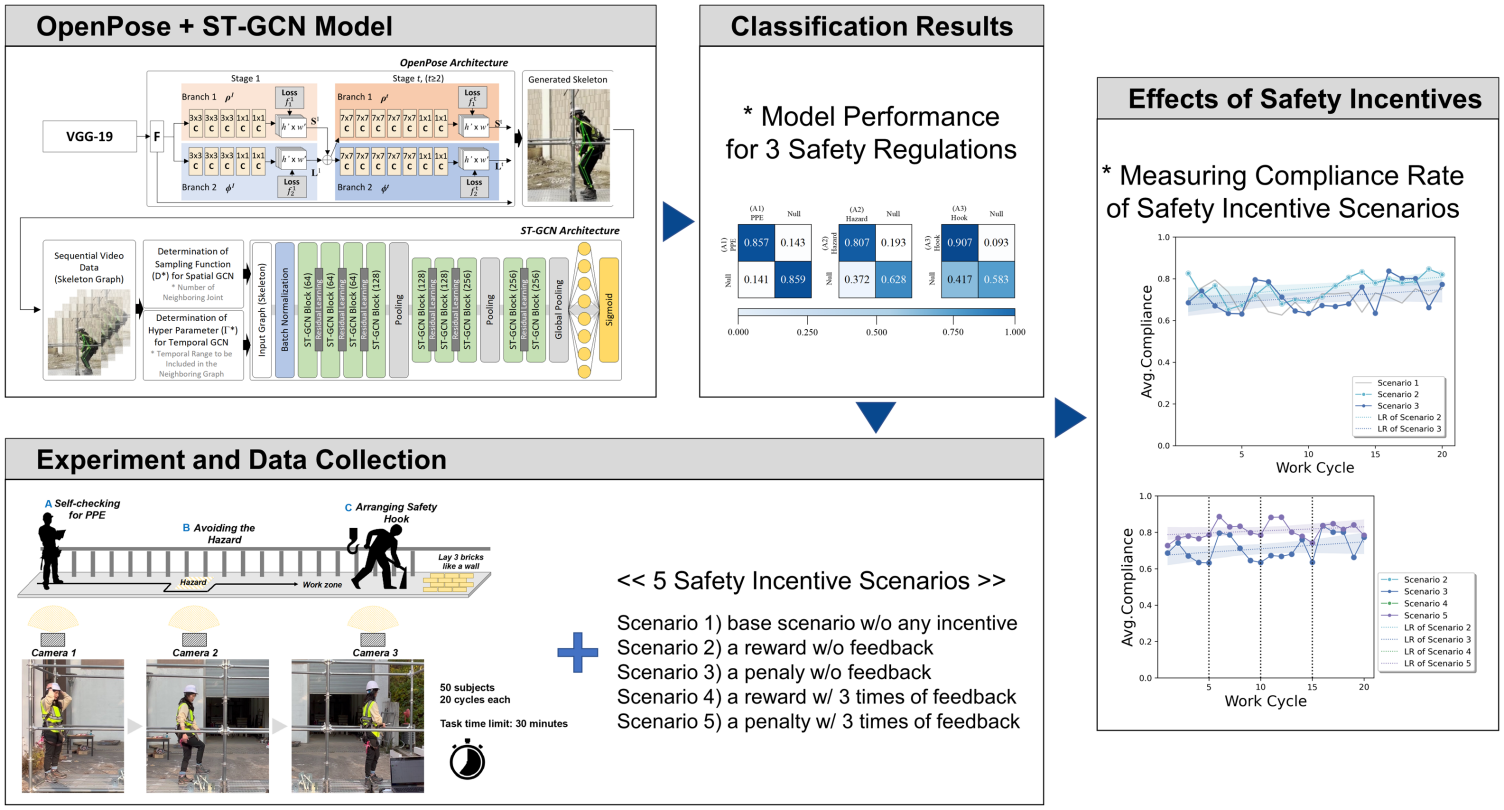
Research framework.

The OpenPose and ST-GCN algorithms can capture human activities from sequences of images by training the spatio-temporal relationships of joints across consecutive frames of images. The OpenPose provides the input images for the ST-GCN with interconnected lines (i.e., bones) and nodes (i.e., joints). Since this approach can recognize the temporal sequences among multiple images, it is capable of recognizing moment-by-moment behaviors. We used the ST-GCN algorithm developed in our previous work (20), modified (adjusting two hyperparameters) to determine safety compliance from sequential frames of the subjects’ activities, to quantitatively measure the effects of safety incentive scenarios. Figure 1 illustrates the research framework used in this study.

### Research questions about the effects of incentives on safety behavior

The main experiment was conducted to test the influence of rewards and penalties on safety behavior. However, previous research has yielded conflicting results depending on the context (i.e., type and duration of work) in which the behaviors are performed. The purpose of this study is to determine the effect of the type of incentives provided to workers on safety behaviors that should be continuous and short term. According to the existing research on human behavior, an individual’s behavior can also be influenced by feedback about their own behavioral state (72–74). This study investigates the effect of providing feedback for improving the safety compliance of workers with varying frequencies of feedback. Three research questions were derived to investigate the effects of the types of incentive programs (i.e., reward and penalty) and the frequency of feedback to workers on safety compliance rates.

Question 1 (Q1) Which type of incentive program-reward or punishment-is more effective in achieving greater compliance with construction safety regulations for construction work?

Question 2 (Q2) Does reminding workers to comply with safety regulations have a greater effect on achieving compliance compared to the case without reminders?

Question 3 (Q3) When giving workers feedback on their compliance with safety regulations, which type of incentive program—reward or punishment—is more effective in achieving greater compliance?

To answer these research questions, we designed five different types of experimental safety incentives by varying the type of incentive and the frequency of feedback.

### Experimental setting

**Figure 2:**
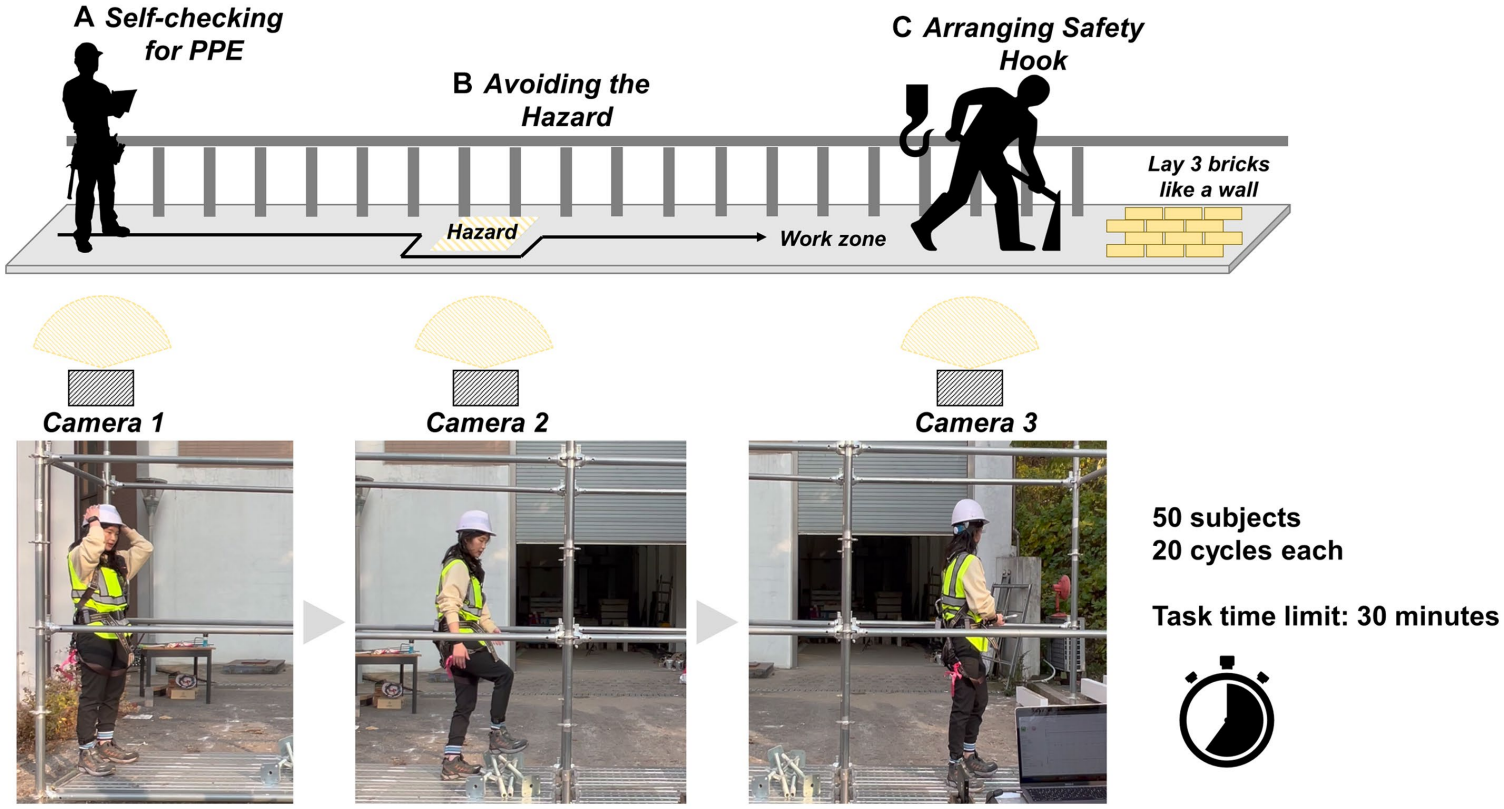
Experimental setting.

We conducted experiments at a construction site to evaluate the different safety incentive scenarios we designed. We recruited 50 healthy construction workers with more than 1 year of work experience. We recorded their work processes using video cameras (iPhone 12) that were installed at each hazard location, as shown in Figure 2. The recorded videos were used to capture the compliance status of the participants and assessed by experts. Information on the participants can be found in Table 1.

**Table 1:** Subject information.

Parameters	Height (cm)	Weight (kg)	Age (y)
Mean	173.5	73.6	27.9
Median	174	74.5	29
Standard deviation	6.78	8.82	4.86
Min. value	163	58	20
Max. value	185	88	35

Video recordings were used to determine the level of compliance of safe behaviors, validate the process by experts with expertise in safety regulations, and confirm the results. To determine compliance with safety regulations and measure behavioral change based on the moment-by-moment safety behavior of workers, we defined the experimental work cycle as relatively short-term (i.e., the average length of a work cycle: 1 ~ 2 min) which includes the essential movements and required safety regulations of the bricklaying tasks. In addition, we designed a controlled environment that reflected the essential characteristics of a construction site including noise and time pressure. To observe how workers complied with safety regulations according to the incentive scenarios, the subjects were asked to repeatedly lay three bricks on the scaffold. We asked the subjects to lay a total of 60 bricks like a wall in the work area, as shown in Figure 2. Thus, they laid three 3 bricks at once and repeated the task 20 times. To create the closest possible workspace environment, the participants had to complete the brickwork within 30 min (i.e., they were under a normal time pressure). Subjects were informed of each safety regulation that they were expected to follow during their tasks. As shown in Figure 2, the subjects started at the first location while checking their personal protective equipment (PPE) themselves (i.e., whether they were wearing their safety helmet and hook properly). Then they make a detour to the hazard installed on the floor. After passing the danger zone, they should properly attach their safety hooks to the scaffold before laying the bricks. For these three safety behaviors, detailed definitions were defined for the experiment. Table 2 shows the safety behaviors and the corresponding regulations that should be complied with by subjects.

**Table 2:** Three safety behaviors and their regulations.

Safety behavior	Definition
Self-checking for PPE	Tap the safety helmet twice with both hands and check whether the safety belt is properly located at the waist.
Avoding the hazard	Stop and make a detour when approaching the hazard installed on the floor.
Arranging the safety hook	Fasten the safety hook to the scaffold.

Through this experimental design, we captured the behavioral changes that occur in moment-by-moment terms and compliance with safety regulations (51, 72, 75–77). The validity of experimental design as a safety behavioral intervention was limitedly confirmed for short-term levels of behavior under the consultation of experts with expertise in construction safety regulations (six safety engineers in construction companies).

### Designing safety incentive scenarios

To answer the proposed research questions and conduct empirical studies on the effects of safety incentives, we designed five scenarios based on general knowledge about the effects of incentives on human behavior (36, 44–47, 78). Subjects performed three safe behaviors per task cycle at each location on the scaffold. In addition, participants were notified that they would receive monetary rewards or penalties depending on their rate of compliance with the safe behaviors, and that they should do their best to achieve perfect compliance.

We created two categories of incentive scenarios with different (1) types of incentives and (2) frequency of feedback on safety compliance by the workers themselves. To create different types of incentives, we designed reward and penalty incentive scenarios based on workers’ compliance with safety regulations. Based on existing studies (32, 37, 41, 42, 79) showing that financial benefits have greater effects than other types of incentives, we defined the content of rewards and penalties as immediate giving or taking of money depending on the compliance rate. In the case of rewards, subjects would start with a credit balance of zero and receive one dollar for each safety compliance. At the end of the experiment, participants received 10 dollars for their tasks if they completed them on time. In penalty cases, the subjects initially received 60 dollars (i.e., the allotted amount for perfect compliance) and had one dollar deducted for each failure to comply with safety regulations. Thus, all subjects who participated in the incentive scenarios received the same amount of money (i.e., 70 dollars), whether it was a reward or a penalty, if they complied perfectly and on time with all regulations during their tasks. To compare the effects of each incentive scenario, a baseline scenario was designed with no incentive intervention. The baseline scenario was that participants received 70 dollars if they completed their twenty tasks on time. To create some time pressure, we also told subjects that if they could not complete their task on time (i.e., within 30 min) they would be deducted five dollars. Thus, subjects had to comply with the safety regulations 60 times during their 20 cycles of the given tasks.

To vary the frequency of feedback on workers’ compliance with safety regulations, we defined two cases: (1) no feedback and (2) feedback provided three times during the 20 cycles of given tasks. When providing feedback to workers, we informed them of their past compliance rate and current account balance (i.e., reward and penalty outcomes). In the case of three-time feedback, we informed participants of their current compliance rate and account balance after the 5th, 10th, and 15th cycles. Table 3 summarizes the designed safety incentive scenarios.

**Table 3:** Summary of the designed safety incentive scenarios.

Scenarios	Type of incentive	Feedback	Definitions
Scenario 1 (base)	–	–	A base scenario without intervention.
Scenario 2	Reward	X	An intervention scenario in which a reward is given without feedback on the subject’s safety behavior.
Scenario 3	Penalty	X	An intervention scenario in which a penalty is given without feedback on the subject’s safety behavior.
Scenario 4	Reward	O	An intervention scenario in which a reward is given with three times feedback on the subject’s safety behavior.
Scenario 5	Penalty	O	An intervention scenario in which a penalty is given with three times feedback on the subject’s safety behavior.

Five scenarios were developed in which the methods used to create incentives varied. Scenario 1 (S1) was defined as a baseline scenario with no incentives or interventions. Scenario 2 (S2) included a reward without feedback and Scenario 3 (S3) included a penalty without feedback. Scenario 4 (S4) included a reward with three times feedback and Scenario 5 (S5) included a penalty with three-times feedback. The results of S1, S2, and S3 were compared to answer the first research question (Q1), which concerned the effects of different types of incentives on safe behavior. The results of the comparison between S2 and S4 or S3 and S5 can answer the second research question (Q2), which is related to the effects of the frequency of feedback about ones’ own compliance status on workers’ safety behaviors. The third question (Q3) was answered by comparing the results of S2 and S3 or S4 and S5.

### Determining workers’ compliance with safe behaviors

As mentioned earlier, each of the 50 participants performed the experiment 20 times. Therefore, 1,000 video records were collected for each safety behavior. As there were three types of safety behaviors in the experiment, the total number of records collected was 3,000. Before classifying the datasets, we edited the videos to include only actions related to the safety regulations of the corresponding spot (i.e., removing scenes of walking from one spot to the next). The videos used in this study were analyzed in AVI format with a frame rate of 30 fps and a resolution of 532 × 300. The average duration of the videos and single activities for each subject were 14.7 s and 3.5 s, respectively. After collecting the data, six safety engineers rated whether the workers followed the safety regulations. Based on the definition of each safety behavior in Table 2, the engineers determined the compliance of each video. For example, the compliance of ‘self-checking for PPE’ was classified as ‘complied’ when all engineers can agree with the presence of activity by tapping the safety helmet and checking the safety belt located at the waist. Table 4 summarizes the results of the data provided by the six safety engineers.

**Table 4:** Results of the video data labeled by experts.

Labeled data	Self-checking for PPE	Avoiding the hazard	Arranging the safety hook
Number of participants following the safety regulations	872	857	933
Number of participants not following the safety regulations	128	143	67
Percentage of participants following the safety regulations	87.2	85.7	93.3

In this study, we used the OpenPose and ST-GCN algorithms to determine subjects’ compliance with safety regulations. Based on the results of a previous study (20) that showed the feasible accuracy (i.e., 0.883 for average F1 score) of the ST-GCN algorithm in identifying the compliance of workers with safety regulations, this methods was also used in this study to automatically detect subjects’ compliance.

In the process of identifying subjects’ compliance, the joints and skeletons of each subject from the OpenPose model were used as inputs to the ST-GCN algorithm. The OpenPose model used in this study applies the Visual Geometry Group (VGG)-19 algorithm (80, 81) to create a feature map. After obtaining sets of sequential images with joint and skeletal information from OpenPose, the ST-GCN algorithm classifies compliance with safety regulations. To implement the ST-GCN algorithm, the sampling function (D) and hyperparameter (Γ) were determined based on an exploratory method with the highest average F-1 scores, *D* = 1 and *Γ* = 6. The framework and process of the ST-GCN algorithm developed in previous studies (20) are shown in Figure 1. To train the model and ensure performance, the collected datasets were split into training and testing datasets of 75% and 25%, respectively. The algorithm determines the compliance (i.e., binary classification) of the subjects with the safety regulations at three hazard points of each task cycle. Based on the feasibility of the algorithm established in previous work (20), this study also evaluates the performance of the algorithm under the conditions of this study (i.e., self-checking for PPE, avoiding the hazard, and attachment of a safety hook) by comparing its accuracy with the expert identification results. The performance of the algorithm was evaluated in terms of accuracy, precision, recall, and F1-score. To measure the effect of each incentive scenario, the algorithm calculates the compliance rate of each subject of each work cycle according to the following steps.

1. A worker is required to comply with three safety regulations, and the algorithm calculates the score as 1 if he/she complies with each regulation; otherwise being calculated as 0.
2. For each work cycle for a worker, the average compliance rate is calculated for the compliance of three regulations (i.e., the average compliance rate for a worker for each cycle ranges from 0 to 1).
3. To present the average compliance rate according to the work cycle, the average compliance rate of all workers for each work cycle is calculated for each scenario.

## Results

### Performance of the determination model

**Figure 3:**
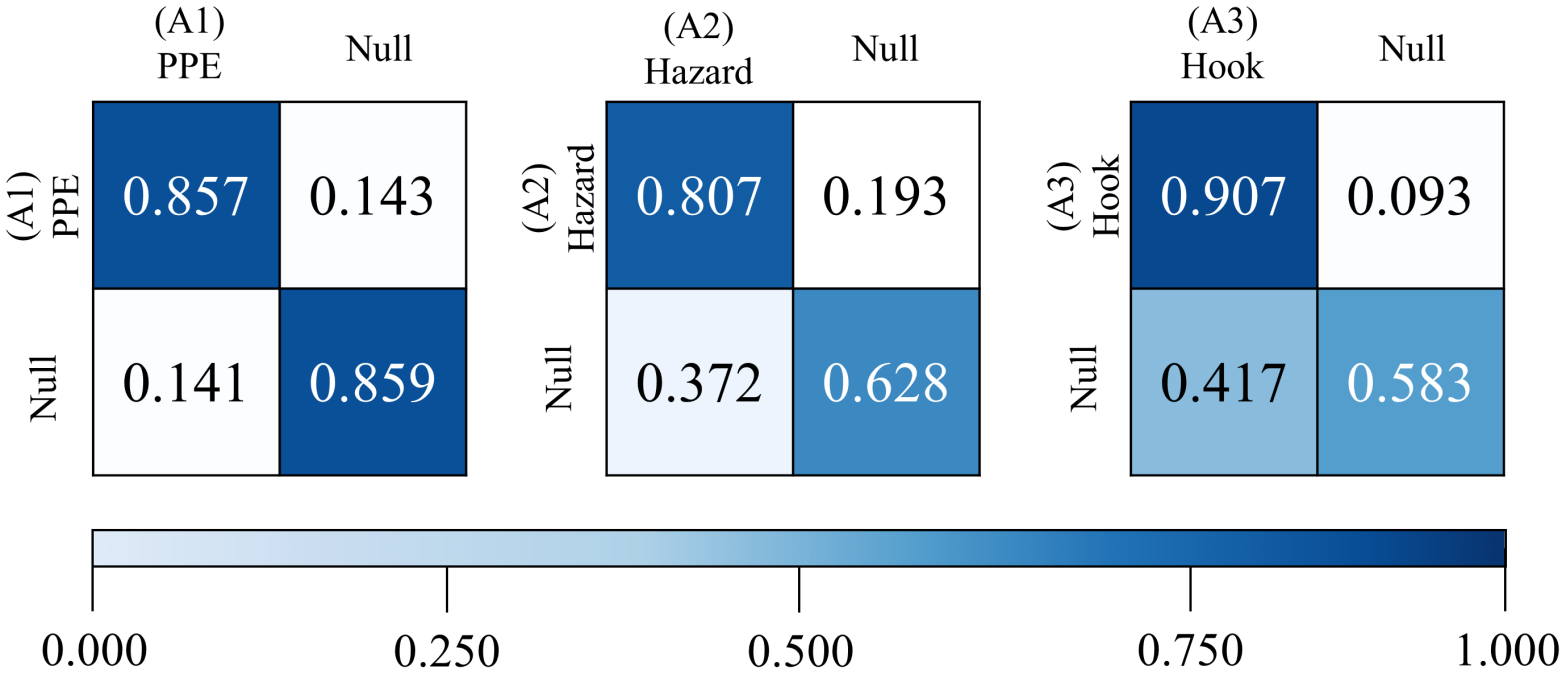
Confusion matrices of classification of the three types of safety behaviors.

An integrated algorithm of OpenPose and ST-GCN was implemented to determine the appropriateness of the three types of safe behaviors. Figure 3 shows the confusion matrices for each safe behavior. We derived these matrices by comparing the results of the algorithm with the determination results of safety experts, which were applied as the ground truth. Table 5 lists the evaluation results for the proposed algorithm. The accuracy values for identifying the three types of behaviors were all greater than 0.8. The case of avoiding hazards showed relatively lower accuracy among the three cases due to false-negatives, such as avoiding the hazard by moving very little. The recall values were also greater than 0.8 and the precision values were greater than 0.9 for all three behaviors. The F1-score of all three behaviors was also greater than 0.85, demonstrating the feasibility of the algorithm in capturing workers’ non-compliance with safety regulations. As for the performance of the algorithm according to the type of behavior, attaching the safety hook showed the highest values for accuracy, recall, and F1-score. The performance scores showed high accuracy, recall, and F1-score in the order of ‘attaching the safety hook,’ ‘self-checking the PPE,’ and ‘avoiding the hazard.’ Despite the behavior of ‘avoiding the hazard’ being newly added in this study, these performance results are consistent with the results of our previous study (20) on the performance of the integrated algorithm of OpenPose and ST-GCN in capturing workers’ behaviors in the video data. Additionally, the approach of this study was evaluated in aspects of classification performance compared with another existing algorithm [Convolutional Neural Networks—Long Short-Term Memory (CNN-LSTM)] run on our experimental datasets in Table 6. The approach of this study shows higher accuracy than existing models in the performance of classification of moment-by-moment safety behaviors.

**Table 5:** Results obtained from the performance metrics of the five types of safety behaviors.

Evaluation metric	Self-checking PPE	Avoiding hazard	Arranging safety hook
Accuracy	0.878	0.825	0.884
Recall	0.858	0.807	0.907
Precision	0.961	0.954	0.966
F1 score	0.907	0.874	0.936

**Table 6:** Results comparing the approach of this study and existing models run on experimental data.

Classification results		CNN-LSTM	Proposed method (based on ST-GCN)
Self-checking for PPE	Accuracy	0.765	**0.878**
	F-1 score	0.847	**0.907**
Avoding the hazard	Accuracy	0.787	**0.825**
	F-1 score	0.853	**0.874**
Arranging the safety hook	Accuracy	0.823	**0.884**
	F-1 score	0.911	**0.936**

Bold indicates the best results.

### Compliance rate according to incentive scenarios

Based on the feasibility of using the algorithm to determine workers’ behavior for proper compliance, we calculated the workers’ compliance rate with safety regulations in our experiment. To quantitatively measure the effect of safety incentive scenarios on worker compliance with safety regulations, we compared the compliance rate of each scenario based on the results from the OpenPose and ST-GCN algorithms.

**Figure 4:**
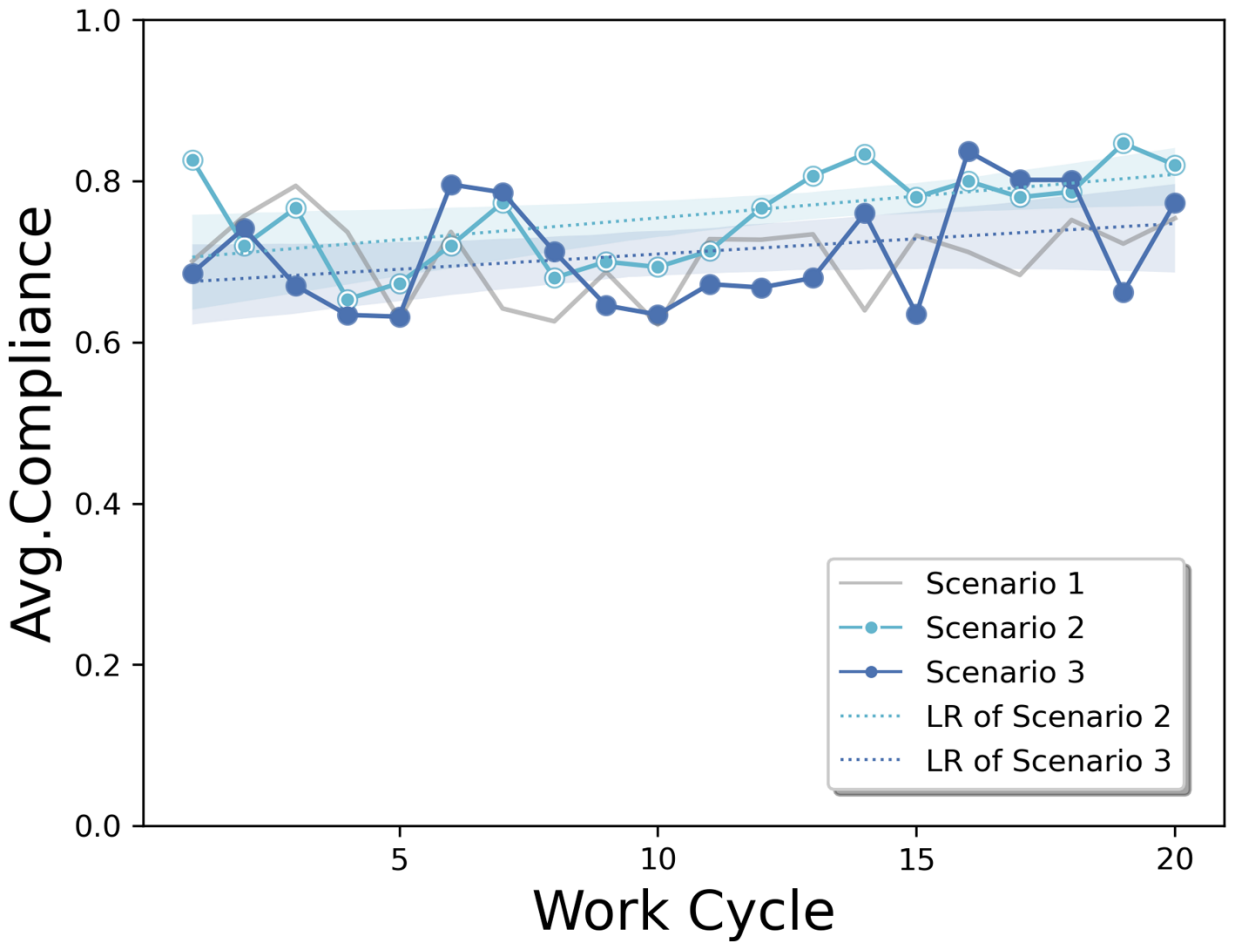
Average compliance rate trends representing the effects of rewards and penalties, including Linear Regression (LR).

Based on Q1, Figure 4 depicts the average compliance rate of subjects who were provided with a reward or penalty, each with a comparison with the case of no incentives (Scenario 1). The average compliance value across the entire work cycle was slightly higher in scenario 2 (0.757) than in scenario 3 (0.711). The linear regression graph (LR) shows that providing a reward to the subjects (Scenario 2) resulted in a higher compliance rate trend than providing a penalty (Scenario 3) throughout the entire work cycle. The average compliance rate in both scenarios increased as the work cycle progressed. The linear slope in scenario 2 (*α* = 0.005, *p* = 0.013) was slightly greater than that in scenario 3 (*α* = 0.004, *p* = 0.160). Both increasing trends throughout the cycle show a higher effect than in Scenario 1, which is the case without safety incentives for the subjects. This implies that providing any safety incentives would have a positive effect on compliance rate improvement. Based on these results, providing a reward has a greater effect than providing a penalty for improving the compliance rate with safety regulations in our experiment.

**Figure 5:**
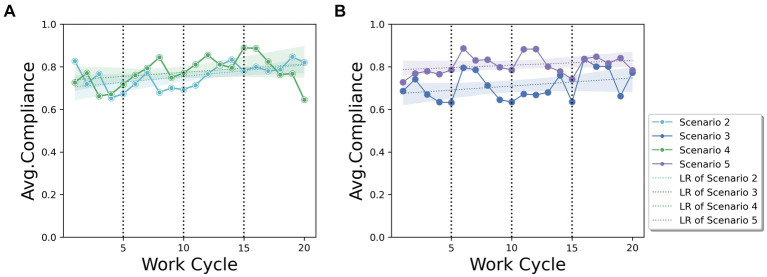
Average compliance rate trends representing the effect of feedback frequency with **(A)** reward and **(B)** penalty, including Linear Regression (LR).

Based on Q2, Figure 5 depicts the average compliance rate trends when providing feedback to the participants about their compliance rate. According to the two types of incentives, the average compliance rates are compared for each type; Figure 5A presents the comparison results of the no-feedback case (Scenario 2) and three feedback cases (Scenario 4) in a rewarding scenario. Figure 5B presents the comparison results of the two different feedback cases (Scenarios 3 and 5) in a penalty scenario. As expected, both the reward and penalty scenarios of providing feedback three times (S4:0.776, S5:0.808) showed a higher compliance rate than the no feedback cases (S2:0.757, S3:0.711). In particular, the average value of the compliance rates for both rewards and penalties shows a clear increase immediately after the feedback cycle (i.e., 5th, 10th, and 15th). Thus, direct feedback to workers regarding their compliance status can motivate them to improve their safety behaviors.

Another finding was that the effect of feedback was more drastic in penalty scenarios than in reward scenarios. As shown in Figure 5A, the two scenarios of providing feedback show similar compliance rates, even though the overall trend of the average compliance rate of providing feedback is slightly higher. Figure 5B shows that providing feedback on the worker’s compliance rate increases the compliance rate compared to the no-feedback case in the penalty scenario. These results imply that the effect of providing feedback to workers of their own compliance on improving the compliance rate would be higher by providing them with a penalty for pointing out the non-compliance status rather than providing a reward. In addition to providing feedback with a penalty, the effect across time shows a different side of providing feedback in terms of rewards and penalties. Both scenarios show slightly increasing trends in compliance rates; however, there was little improvement in feedback with a penalty over the work cycle. This implies that repeated mention of penalties can be less effective than repeated mention of incentives in motivating workers to comply with regulations from a long-term perspective.

**Figure 6:**
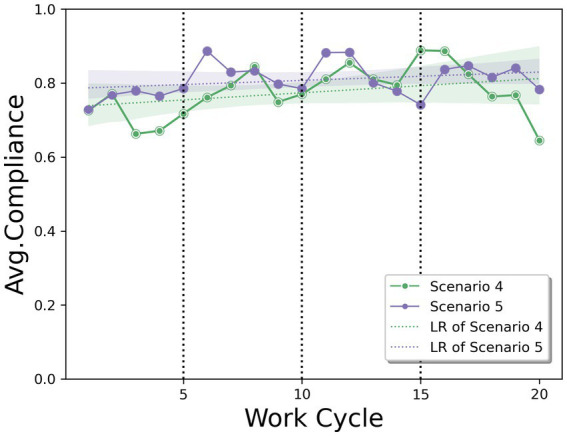
Average compliance rate trends representing the effects of rewards and penalties in the feedback situation, including Linear Regression (LR).

Based on Q3, Figure 6 shows the evolution of the average compliance rate in a reward and penalty scenario, with feedback given three times during the experiment in both cases. Similar to Figure 5, it can be seen that a penalty produces a higher compliance rate than a reward, in all feedback. This result is in contrast to the finding from Q1, which showed a greater effect of a reward without feedback. Thus, the effect of a reward and penalty scenario can be maximized to improve compliance rates depending on whether workers receive feedback on their own behavior. In our experimental setting, providing a penalty with feedback (Scenario 5) resulted in the highest compliance rate with safety regulations.

Moreover, the effect of improving the compliance rate across the work cycle was higher when a reward (*α* = 0.004, *p* = 0.152) was offered than a penalty (*α* = 0.002, *p* = 0.214). This trend is consistent with the findings in Figure 4, which shows scenarios without feedback. These results also support the idea that providing rewards may be more effective in motivating workers to comply with regulations in the long run.

## Discussion

### Implications for the effects of reward/penalty across work cycles

The results derived from Q1 indicate that rewarding workers is more effective than punishing them when there is no feedback on workers’ compliance. This result includes several aspects, with some previous results indicating that punishment was more effective than reward, although the average value of the compliance rate between a reward (S2:0.757) and a penalty (S3:0.711) is not significantly different. The linearly increasing trend of the compliance rate over the work cycle also showed a higher value for a reward (S2:0.005) than for a penalty (S3:0.004). This implies that a positive incentive is more effective within a relatively short period of time (i.e., 20 work cycles in this study) because it exerts less psychological pressure on workers to comply with safety regulations and complete their tasks on time. Subjects who participated in Scenarios 2 and 4 rarely mentioned non-compliance or the deadline after the experiment (5%); however, 35% of the subjects who participated in Scenarios 3 and 5 related their non-compliance and task failure with their perceived pressure.

In contrast, a penalty case is more effective when three feedback points are provided on the workers’ own compliance rate. Despite the psychological pressure that the participants perceived, a higher effect on the compliance rate was presented by being aware of the risk of non-compliance through feedback. Thus, the effect of safety incentive scenarios may change depending on whether workers perceive the penalties as psychological pressure that may lead to mistakes, or whether they are aware of the potential risks that may lead to strict compliance with regulations. These conflicting results leave room for further research on the effects of providing feedback on workers’ motivation behave safely.

### Implications for the effects of feedback with reward/penalty in the extreme case

**Figure 7:**
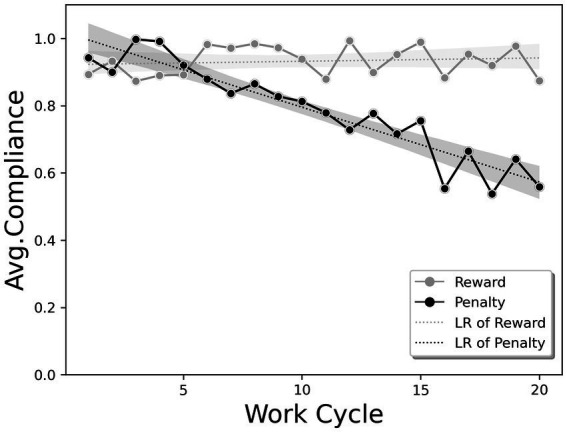
Average compliance rate trends for extreme intervention cases in the form of a reward or penalty, including Linear Regression (LR).

The results of our experiment (Q2), show that providing a punishment is more effective than providing a reward when we give workers feedback on their own compliance rate and their balance. To investigate the reasons for the differences between reward and punishment with feedback, we conducted an additional experiment of an extreme case in which feedback was given to the subjects. The 10 subjects who had participated in Scenario 1 performed the experiment, in which feedback was given in each work cycle. Figure 7 shows the evolution of the average compliance rate for reward and punishment in each feedback cycle. When participants received a reward, they showed a similar average compliance rate throughout the work cycle with a value of 0.932. In contrast, when a punishment was provided, there was a clear downward trend throughout the work cycle, which was particularly steep in the latter cycles, with a value of 0.784.

The difference between the two cases stems from the participants different responses to the constant feedback they received. In the reward case, the constant feedback did not elicit any particular response, whereas in the punishment case, some subjects felt a kind of psychological pressure when they were noticed for their non-compliance and the reward balance deducted. The constant feedback on task non-compliance led to a feeling of giving up rather than motivation to complete the task, and some subjects showed very low compliance rates in the latter cycles of the task. In an interview with these participants, they mentioned that the constant reminder of penalties for their non-compliance, especially at the beginning of the cycles, induced panic. These results were more apparent because of the experimental situations (i.e., not realistic, such as wage cuts in the workplace). Nevertheless, this implies that constantly reminding workers of the negative condition of punishment is not effective in motivating safe worker behavior. Moreover, responses to penalties showed greater individual variance across participants than responses to rewards.

### Limitations and future applications

Because the decision of how to behave is directly related to the perception of the context associated with the corresponding behavior in question (82, 83), the way in which workers accept safety compliance incentives is critical to improving behavior. Awareness of the type or level of incentives may vary from person to person, and the effect of incentives may not show up consistently, even when a common incentive scenario is applied to a large number of workers. In addition, a particular safety incentive scenario, which was selected as the most effective scenario (Scenario 5), may only be effective in relatively short-term applications, such as the experimental conditions in this study. Therefore, each incentive scenario may have different effects in long-term applications, such as over several months. In the long term, workers could become desensitized to incentive scenarios, leading to a decrease in the effectiveness of the measures. A possible negative work climate is another limitation that was not fully investigated in this study. Depending on the level of reward and penalty, or the way feedback is delivered, the incentive scenario may cause side effects such as excessive tension during work or a culture of competition among workers (84–86), rather than having a positive effect on improving behavior.

Based on the results of this study, construction managers should consider the exact conditions for applying incentive scenarios, such as setting long-term and short-term perspectives according to the conditions of the actual work environment. A method of providing individualized feedback to each worker can also be considered while monitoring the compliance rate of individual workers in terms of their response to the appropriate incentive scenario. Considering the automated approach of this study (i.e., determining the compliance with safety regulations using sequential images), a system can be developed that provides individual feedback to workers in nearly real-time through PPEs (e.g., sensors in safety helmets or safety vests). The algorithm for providing personalized feedback can be defined according to the tendencies of compliance rate trends after feedback (i.e., providing penalty feedback for users who are more responsive to penalties when the compliance rate decreases by more than 10% compared to the previous feedback). This will make the safety incentive program more effective than existing ones, which cannot fully consider the various conditions of individuals, such as the degree of risk of the work, the working environment, and personal perception of incentives/penalties.

## Conclusion

The objective of this study was to measure the effect of different types of safety incentives by comparing the average compliance rates of each scenario in experimental settings (i.e., laying bricks on the latter parts of the scaffold while complying with the three types of safety regulations). Using the OpenPose and ST-GCN algorithms, worker compliance rates were calculated in different scenarios with rewards and penalties, with and without feedback. The results showed that each safety incentive had a positive effect on compliance, with a reward having a greater effect than a penalty. The effect of feedback was more significant in the penalty scenarios than in the reward scenarios, and a penalty with feedback resulted in the highest compliance rate with safety regulations. The method used in this study to determine worker compliance with safety regulations can easily capture momentary safe behaviors through video recordings. Therefore, this image-based model can facilitate the design of the safety incentive scenario by focusing more on the safety behaviors themselves, which has improved existing incentive programs that typically focus on outcomes.

This study provides an accurate assessment of safety incentives and their effectiveness using an image-based approach to observe especially workers’ moment-to-moment behaviors and their changes extending the existing works. In aspects of methodologies, the ST-GCN-based algorithms to identify the compliance of workers with safety regulations enable assessing relatively short-momentary behaviors only considering the worker’s sequential poses. This contributes to developing an approach that can determine the finer level of behavioral change that occurs even at the unconscious level. Based on the results of this study, construction managers should consider the exact conditions for applying incentive scenarios, such as setting long-term and short-term perspectives according to the conditions of the actual work environment. A method of providing individualized feedback to each worker can also be considered while monitoring the compliance rate of individual workers in terms of their response to the appropriate incentive scenario.

A possible future study could focus on exploring the long-term effects of safety incentives by using an image-based model to capture workers’ safe behaviors and examine why workers may be more motivated by rewards than penalties in the long run.

## Data Availability

The raw data supporting the conclusions of this article will be made available by the authors, without undue reservation.
